# Testosterone deficiency and chronic kidney disease

**DOI:** 10.1016/j.jcte.2024.100365

**Published:** 2024-08-14

**Authors:** Michael Zitzmann

**Affiliations:** Centre of Reproductive Medicine and Andrology of the University, Domagkstrasse 11, 48149 Münster, Germany

**Keywords:** Testosterone, Testosterone Therapy, Male hypogonadism, Classical Male hypogonadism, Functional Male hypogonadism, Chronic Kidney Disease

## Abstract

Testosterone’s biological functions are extensive, influencing reproductive and systemic health. It plays a vital role in sexual functions, muscle protein synthesis, bone metabolism, fat distribution, and cardiovascular health. The hormone also affects mood, cognitive function, and erythropoiesis, underscoring its importance in both physical and mental health.

Testosterone deficiency, or male male hypogonadism, is increasingly recognized as a significant health issue affecting various bodily systems, also in the context of chronic kidney disease (CKD). Recent research indicates a complex interplay between testosterone levels and renal health, suggesting that male male hypogonadism may both impact and be impacted by CKD. The latter is characterized by a gradual loss of kidney function, affects millions globally and is often associated with diabetes mellitus, arterial hypertension, and autoimmune diseases. Men with CKD frequently experience lower testosterone levels, which can exacerbate muscle wasting, reduce quality of life, and increase cardiovascular risk. Overall, low testosterone levels in CKD patients are associated with increased morbidity and mortality.

Several mechanisms explain the relationship between CKD and testosterone deficiency. The uremic environment in CKD disrupts the hypothalamic-pituitary–gonadal axis, impairing hormone production. Nutritional deficiencies and chronic inflammation common in CKD patients further suppress gonadal function. The consequences of low testosterone in CKD are profound, with studies suggesting that testosterone replacement therapy (TRT) might improve clinical outcomes, though the long-term effects and causal relationships remain under investigation.

The potential benefits of TRT in CKD patients might be significant. TRT can enhance muscle mass and strength, address anemia by stimulating erythropoiesis, improve bone density, and possibly offer cardiovascular benefits by improving body composition and insulin sensitivity. General symptoms of male hypogonadism, such as deteriorated psychological, sexual and physical wellbeing, can be improved by TRT. However, these benefits must be weighed against potential risks. TRT may exacerbate fluid retention, arterial hypertension, or exacerbate existing heart failure, particularly in CKD patients with pre-existing cardiovascular comorbidities. Additionally, concerns about the progression of renal disease via several testosterone affected pathways involving renal tubular integrity exist, highlighting the need for careful patient selection and monitoring.

Understanding this relationship is crucial for developing comprehensive treatment strategies that address both renal and endocrine dysfunctions, highlighting the need for integrated patient care, which means good collaboration between subspecialists like nephrologists, endocrinologists, urologists and primary care providers, aiming to improve outcomes and quality of life while mitigating adverse effects.

## Introduction

**Fig. 1:**
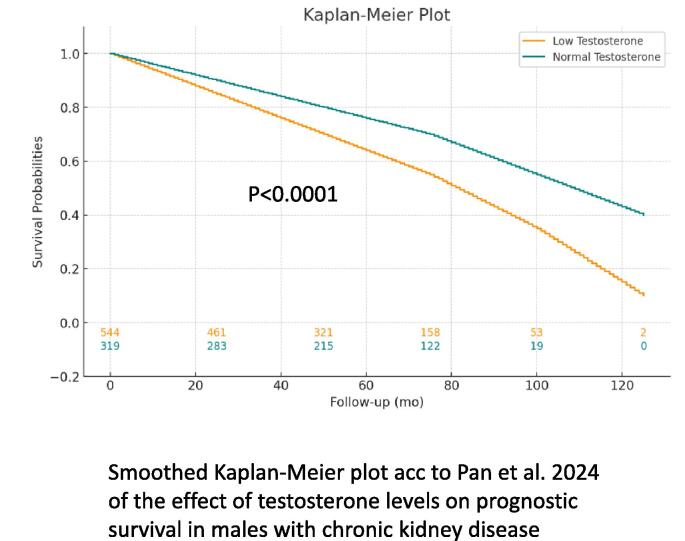
Smoothed Kaplan-Meier plot acc to Pan et al. 2024 of the effect of testosterone levels on prognostic survival in males with chronic kidney disease.

Testosterone deficiency, also known as male hypogonadism, is increasingly recognized as a significant health issue that affects various bodily systems. A complex interplay between testosterone and chronic kidney disease (CKD) has emerged from recent research, suggesting that this endocrine function might significantly impact renal health and vice versa [1], [2] (Fig. 1).

Testosterone, a primary male sex hormone, plays a crucial role in maintaining muscle mass, bone density, and overall energy levels. In the general population, testosterone levels decline with age, albeit this decline is facilitated, precipitated, or exacerbated by chronic health conditions, markedly obesity and/or inflammation, also involving CKD [1], [2]. CKD, characterized by a gradual loss of kidney function over time, affects millions globally and has multiple etiologies including diabetes mellitus, hypertension, and autoimmune diseases [3], [4]. Emerging evidence suggests that men with CKD are more likely to experience lower testosterone levels compared to the general population. This association raises the hypothesis that CKD may lead to or worsen male hypogonadism. Several mechanisms have been proposed to explain this relationship. Firstly, the uremic milieu typical of CKD may interfere with the hypothalamic-pituitary–gonadal axis, disrupting hormone production and regulation. Secondly, nutritional deficiencies, a common issue in CKD, could impair the synthesis of testosterone. Additionally, the chronic inflammation associated with CKD might suppress gonadal function, leading to lower testosterone levels [1], [2].

The consequences of low testosterone in CKD are profound. Testosterone deficiency can exacerbate muscle wasting, reduce quality of life, and increase the risk of cardiovascular diseases, which are already heightened in CKD patients. Moreover, some studies suggest that testosterone replacement therapy may improve various clinical outcomes in CKD, albeit on associations of values in relation to outcomes and longitudinal data of non-interventive nature [1], [3]. Other research speculates that testosterone might accelerate diabetic nephropathy by increasing blood pressure or directly inducing renal tubule cell injury [5], [6], [7]. Despite the growing body of research, many questions remain. The causality and directionality of the relationship between CKD and testosterone deficiency are not fully established, and the long-term effects of testosterone therapy in this population need thorough investigation. As the demographic with CKD ages, understanding these connections will be crucial for developing comprehensive treatment strategies that address not only the renal aspects of CKD but also its systemic manifestations, including endocrine dysfunctions [1].

**Fig. 2:**
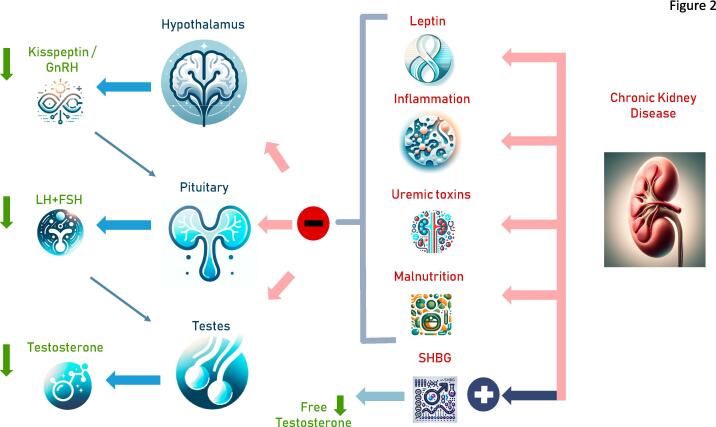
Induction of male hypogonadism by Chronic Kidney Disease. SHBG: sex hormone binding globulin.

This burgeoning field invites a deeper exploration into how hormonal health influences and is influenced by renal function, with implications for both pathophysiology and therapeutic approaches. The interdependence of endocrine and renal health in the context of chronic diseases exemplifies the complexities of internal medicine and the necessity for an integrated approach to patient care. This text aims at exploring the intricate relationships between testosterone deficiency and CKD, underscoring the necessity for a nuanced understanding of their bidirectional dynamics (Fig. 2).

## Biological functions of testosterone in men

Testosterone plays a multifaceted role in orchestrating a diverse range of biological functions vital for sustaining health and well-being. Herewith, the expansive and profound influence of testosterone across various physiological domains, emphasizing its significance in both reproductive and systemic health is delineated. Testosterone is also pivotal in the development of male secondary sexual characteristics during puberty, such as the growth of facial and body hair, voice deepening, and the enhancement of muscle mass and strength. Its role extends to spermatogenesis, where it ensures the production of viable sperm by activation of respective Sertoli cell receptors, and it is crucial in maintaining libido and erectile function, thus underpinning male fertility [2], [8].

In addition, the hormone significantly enhances muscle protein synthesis, thereby augmenting muscle mass and strength. Testosterone also plays a vital role in bone metabolism by promoting bone growth and maintaining bone density, crucial for preventing osteoporosis and sarcopenia, particularly in aging males [9], [10]. Testosterone exerts influence on metabolism including the regulation of fat distribution, favoring a higher muscle-to-fat ratio. It impacts glucose and lipid metabolism, thus affecting the risk and management of metabolic disorders such as type 2 diabetes and dyslipidemia. Its regulatory effects on insulin sensitivity and adipose tissue distribution are essential for the metabolic health of males [11], [12].

Testosterone has complex interactions with the cardiovascular system, affecting endothelial function, cholesterol levels, and blood pressure. While the relationship between testosterone levels and cardiovascular health is intricate, maintaining physiological levels of testosterone may be associated with protective cardiovascular effects, such as enhanced vasodilation and reduced atherosclerotic plaque formation in some populations while does not seem to increase the rate of cardiovascular events in already compromised older men [13], [14]. Furthermore, the hormone modulates mood, cognitive function, and overall psychological and sexual well-being. Low levels of testosterone are linked with an increased risk of depression, cognitive decline, and mood disturbances, highlighting its crucial role in the central nervous system and underscoring its importance not only in physical but also in mental health [15], [8], [2]. Testosterone stimulates erythropoiesis, the production of red blood cells, which is vital for oxygen transport and aerobic capacity. This effect is mediated through erythropoietin and has significant implications for anemia management in men, particularly those with chronic illnesses or those undergoing androgen deprivation therapy [16], [17].

Through its extensive and critical roles, the importance of testosterone transcends reproductive functions, underscoring its pivotal role in the holistic management of male health. An in-depth understanding of these diverse biological functions is indispensable in diagnosing, treating, and managing conditions related to testosterone deficiency [2].

## Overview of testosterone synthesis and its regulation

Testosterone synthesis and its regulation within the male body represent a complex, intricately controlled endocrine process, fundamental to understanding both normal physiological functions and the pathophysiology of various disorders. The process of testosterone production primarily takes place in the Leydig cells of the testes, under the meticulous control of the hypothalamic-pituitary–gonadal (HPG) axis, showcasing a classic example of endocrine regulation through feedback mechanisms.

The synthesis of testosterone is initiated by the pulsatile release of gonadotropin-releasing hormone (GnRH) from the hypothalamus, which stimulates the anterior pituitary gland to secrete luteinizing hormone (LH). LH then acts on the Leydig cells, triggering a cascade of enzymatic reactions that convert cholesterol, the substrate for all steroid hormones, into testosterone through a series of steps involving the enzyme cytochrome P450 side-chain cleavage enzyme (P450scc) and subsequently other enzymes such as 3β-hydroxysteroid dehydrogenase (3β-HSD), 17α-hydroxylase/17,20-lyase (CYP17A1), and 17β-hydroxysteroid dehydrogenase (17β-HSD) [15]. This biosynthesis of testosterone is a multistep process that involves the conversion of cholesterol into pregnenolone, followed by the sequential transformation into progesterone, 17α-hydroxyprogesterone, androstenedione, and ultimately testosterone. Notably, the rate-limiting step in this pathway is the transport of cholesterol into the mitochondria, facilitated by steroidogenic acute regulatory protein (StAR), underscoring the critical role of cholesterol availability and intracellular transport in testosterone synthesis [15].

Regulation of testosterone production is achieved through negative feedback mechanisms where elevated levels of testosterone exert an inhibitory effect on the hypothalamus and pituitary, reducing GnRH and LH secretion, respectively, to maintain homeostasis. Additionally, testosterone can be locally converted to dihydrotestosterone (DHT) by 5α-reductase isoenzymes in certain tissues or aromatized to estradiol in adipose tissue, which also partakes in feedback regulation of the HPG axis, demonstrating the intricate balance and interplay of androgens and estrogens in male physiology [15]. Testosterone levels decline with advancing age, this mostly being a function of the general health status of the male [18], [19].

Understanding the nuances of testosterone synthesis and its regulation is essential for not only diagnosing and treating disorders related to androgen deficiency or excess but also appreciating the broader implications of testosterone in metabolic, cardiovascular, and overall health.

## Etiology of testosterone deficiency in men

Testosterone deficiency, also known as male hypogonadism, can arise from a variety of etiologies, broadly categorized into primary (testicular) and secondary (hypothalamic-pituitary) causes [2]. Understanding these underlying factors is crucial for the diagnosis, management, and treatment of testosterone deficiency.

Primary male hypogonadism is characterized by underactive testes that do not produce adequate levels of testosterone, despite normal or elevated levels of pituitary gonadotropins (LH and FSH). The causes include several pathologies. Klinefelter syndrome as a genetic condition, characterized by an extra X chromosome (47,XXY), remains a common cause. Men with Klinefelter syndrome often present with primary male hypogonadism, leading to reduced testosterone production [20]. Another notable cause for primary male hypogonadism is cryptorchidism. Another contributory factor is orchitis, which refers to inflammation of the testes, often sparked by viral infections like mumps. This inflammation can wreak havoc on the Leydig cells, the very cells tasked with testosterone production. Additionally, physical injuries to the testes disrupt testosterone synthesis [2].

Secondary male hypogonadism points to problems beyond the testes, specifically involving the hypothalamus or the pituitary gland. These conditions hinder the production of luteinizing hormone (LH) and follicle-stimulating hormone (FSH), which are crucial for stimulating testosterone production in the testes. Various factors can lead to such disruptions. Pituitary tumors or diseases, particularly benign adenomas, can impede the pituitary gland's hormone production capabilities [2]. Certain genetic disorders, such as Kallmann syndrome and congenital hypogonadotropic male hypogonadism, are linked with GnRH deficiency, leading to delayed or absent puberty and associated testosterone deficiencies [2].

Furthermore, the concept of functional male hypogonadism has been identified, distinguishing itself as a potentially reversible condition, closely associated with comorbidities such as obesity, type 2 diabetes, or severe illness. Hyperprolactinemia and also CKD are classified as causes for functional male hypogonadism [2], [21]. Unlike other forms of male hypogonadism, this type does not originate from defects in the testes or the HPG axis but from external factors such as the increased secretion of leptin and inflammatory cytokines by visceral fat, along with an augmented conversion of testosterone to estradiol. This increased level of estradiol further suppresses the secretion of gonadotropins, compounding the problem.

Obesity and related metabolic disorders present the paramount avenue through which functional male hypogonadism can manifest. The excessive adipose tissue in obese individuals functions as an endocrine organ, releasing hormones and cytokines that disrupt the hypothalamic-pituitary–gonadal (HPG) axis, consequently reducing testosterone levels [11], [21].

## Diagnosis and treatment options regarding testosterone deficiency

The diagnosis and treatment of testosterone deficiency, or male hypogonadism, involve a combination of clinical assessment, biochemical tests, and personalized therapeutic approaches [2], [21].

## Diagnosis of testosterone deficiency

The process of diagnosing testosterone deficiency begins with a meticulous clinical evaluation, which includes a detailed clinical history and a comprehensive physical examination. This step is crucial as it helps to pinpoint symptoms indicative of testosterone deficiency. Such symptoms might include reduced libido, erectile dysfunction, fatigue, loss of muscle mass and strength, increased body fat, decreased bone density, and mood disturbances. Gathering a thorough medical history is also essential as it provides insight into potential causes like chronic illnesses, the impact of certain medications, or lifestyle factors that might be contributing to lowered testosterone levels. Following the clinical evaluation, the diagnostic journey continues with laboratory tests, which are pivotal in confirming testosterone deficiency. The Endocrine Society advocates for the measurement of morning total testosterone levels using a reliable assay as the primary diagnostic test. This recommendation is based on the natural diurnal variation of testosterone, which typically peaks in the morning [2], [21]. If the initial total testosterone reading is low or borderline, it is advisable to conduct a repeat measurement of total testosterone. At this stage, assessing free or bioavailable testosterone levels is also crucial. For greater accuracy, methods like calculated free testosterone or equilibrium dialysis are preferred.

To further refine the diagnosis, the levels of serum luteinizing hormone (LH) and follicle-stimulating hormone (FSH) are evaluated. This assessment is vital as it helps distinguish between primary male hypogonadism and secondary male hypogonadism. Depending on the outcomes of the initial evaluations, additional assessments may be necessary. These could include measuring prolactin levels, conducting thyroid function tests, performing iron studies, and potentially undertaking pituitary imaging. These tests are integral in ruling out other conditions that might either mimic the symptoms of testosterone deficiency or contribute to them in some way. Each step in this evaluation process is guided by the goal of accurately identifying and understanding the underlying causes of testosterone deficiency to ensure that appropriate and effective treatments can be administered [2], [21].

## Treatment options for testosterone deficiency

The management of testosterone deficiency is focused on alleviating symptoms, enhancing quality of life, and addressing any underlying causes that may contribute to the condition. Various treatment options are available, each tailored to the specific needs and circumstances of the individual. One of the primary treatments is Testosterone Replacement Therapy (TRT), which can be administered through several methods: intramuscular or subcutaneous injections, transdermal gels, or subdermal pellets. The choice of TRT method depends on factors such as patient preference, the pharmacokinetics of the preparation, and cost considerations. The objective of TRT is to restore testosterone levels to the normal physiological range, thereby relieving the symptoms commonly associated with male hypogonadism [2], [21].

In cases where secondary male hypogonadism is caused by disorders of the pituitary gland or hypothalamus, treating the underlying condition is essential. This could involve surgical interventions to remove pituitary tumors or medical management of other chronic conditions that are contributing to the hormone imbalance, i.e., prolactin-lowering medications such as cabergoline [2], [21].

Lifestyle modifications also play a critical role in managing testosterone deficiency, especially in functional male hypogonadism. Conditions like obesity, metabolic syndrome, and type 2 diabetes are known to negatively affect testosterone levels. Addressing these through weight loss, improved diet, and increased physical activity not only boosts testosterone levels but also improves overall health [2], [21].

Regular monitoring and follow-up are crucial for patients undergoing TRT. This ongoing supervision helps to ensure that testosterone levels and symptoms remain in check and allows for the timely identification and management of any potential side effects. Key aspects of monitoring include checking hematocrit levels, lipid profiles, liver function, and prostate health through digital rectal exams and prostate-specific antigen (PSA) testing [2], [21].

In situations where TRT is not indicated, mainly when the patient has a wish for paternity (TRT suppresses sperm production by blocking the secretion of gonadotropins), alternative therapies such as clomiphene citrate, an estrogen receptor modulator, may be used. Other substances acting on the estradiol-pathway are tamoxifen as another receptor modulator or anastrozole as an aromatase inhibitor. These substances will only work in cases of a functional hypothalamic-pituitary and are off-label. They might be given on compassionate care grounds. It has to be mentioned that blocking the estrogen signal might result in long-term loss of bone density. The use of gonadotropins such as hCG, which can bind to the LH-receptor and recombinant FSH is hence there the gold standard in treating male hypogonadism in men who seek paternity [2], [21].

Overall, the approach to treating testosterone deficiency is highly individualized, considering factors like the severity of symptoms, the underlying causes, and patient preferences. The field continues to evolve with ongoing research, which is aimed at refining treatment strategies to optimize outcomes for men affected by male hypogonadism.

## A short overview and introduction to chronic kidney disease (CKD)

Chronic kidney disease (CKD), a condition characterized by a gradual loss of kidney function over time, stands as a global public health concern, affecting approximately 10 % of the world's population [22], [23]. The kidneys' role in filtering wastes and excess fluids from the blood, crucial for maintaining the body's electrolyte balance and blood pressure, becomes compromised in CKD, leading to the accumulation of harmful substances in the body. The etiology of CKD is multifaceted, with diabetes and hypertension being the leading causes, underscoring the disease's connection to broader metabolic and cardiovascular disorders [24], [23]. The progression of CKD is silent and insidious, often culminating in end-stage renal disease (ESRD) which necessitates life-sustaining dialysis or kidney transplantation. Given its stealthy advancement and significant impact on mortality and morbidity, CKD's prevalence underscores an urgent need for early detection and intervention strategies aimed at curbing its rise and mitigating its impacts on the global healthcare system [25], [23].

## Etiologies of CKD

Chronic kidney disease (CKD) emerges as a consequence of a variety of systemic diseases and localized renal conditions that deteriorate kidney function over time. A nuanced understanding of these etiologies is pivotal for devising effective management and treatment strategies. Diabetic nephropathy stands as the foremost cause of CKD globally, primarily due to the long-term impacts of diabetes mellitus on kidney function. High blood sugar levels in diabetic patients lead to significant changes within the kidneys, such as glomerular hyperfiltration, thickening of the glomerular basement membrane, and eventual scarring known as glomerulosclerosis. Preventive measures are centered around strict control of blood sugar levels and managing hypertension to mitigate these effects [26], [23].

Hypertensive nephrosclerosis also ranks high among the causes of CKD, especially prevalent in African American populations. Persistent high blood pressure inflicts damage on renal blood vessels, diminishing kidney function. Therefore, controlling blood pressure is critical to slowing the progression of CKD due to hypertension [27]. Glomerulonephritis, which involves inflammation of the glomeruli, can be either acute or chronic and serves as a major cause of CKD. This condition may arise from autoimmune disorders, infections, or remain idiopathic. Treatment typically targets the underlying cause and may involve the use of immunosuppressive drugs [28].

Polycystic Kidney Disease (PKD), a genetic ailment, is characterized by the development of numerous cysts in the kidneys which lead to kidney enlargement and impaired function. The management of PKD focuses on controlling hypertension and alleviating symptoms, with end-stage cases often requiring dialysis or kidney transplantation [29], [23]. Tolvaptan is a significant therapeutic agent in the management of PKD. It functions as a selective vasopressin V2-receptor antagonist, which means it inhibits the action of vasopressin, a hormone that plays a key role in cyst growth and kidney volume expansion in PKD. By blocking vasopressin, tolvaptan slows the increase in kidney size and the progression of kidney dysfunction over time in patients with autosomal dominant PKD. However, it is not without side effects; common ones include thirst, polyuria (increased urine production), and liver enzyme elevations, which necessitate careful monitoring of liver function during treatment. Tolvaptan offers a targeted approach, providing a specific treatment option that addresses the pathophysiological processes underlying PKD progression [30].

Interstitial nephritis, another factor, entails inflammation of the kidney’s tubules and surrounding structures. It may be triggered by drugs, autoimmune diseases, or infections. While acute interstitial nephritis might be reversible with the removal of the causative agent or treatment of the underlying disease, chronic forms can result in permanent kidney damage [31]. Obstructive uropathy, caused by blockages in the urinary tract such as kidney stones, tumors, an enlarged prostate, or anatomical anomalies, creates back pressure in the kidneys that harms renal tissue. The treatment focuses on removing the obstruction to alleviate pressure and prevent further damage [32], [23]. Renal vascular diseases like renal artery stenosis, which narrows the renal arteries, diminish blood flow to the kidneys and can culminate in CKD. Management typically involves blood pressure control and procedures to reestablish renal blood flow [33], [34].

Additional risk factors such as smoking, obesity, and exposure to nephrotoxic substances like certain drugs or heavy metals also play a significant role in the development and progression of CKD. Preventive strategies include lifestyle adjustments and avoiding nephrotoxic agents [35].

Managing CKD effectively entails addressing these underlying causes through various strategies that range from medical interventions, like controlling blood pressure and sugar levels, to lifestyle changes and, when necessary, surgical interventions. These measures are essential to slow the progression of the disease and improve quality of life for those affected [23].

## Special emphasis on Auto-Inflammatory causes of chronic kidney disease

Autoimmune diseases contribute significantly to the etiology of chronic kidney disease (CKD) by triggering inflammation and damage in various parts of the kidneys. These conditions can lead to both acute and chronic forms of kidney injury, ultimately affecting renal function and contributing to the progression of CKD. Among the autoimmune diseases that can impact kidney health, systemic lupus erythematosus (SLE) and rheumatoid arthritis (RA) are prominent.

Systemic lupus erythematosus (SLE), a chronic autoimmune disease characterized by systemic inflammation affecting multiple organ systems, can lead to lupus nephritis (LN), one of the most serious complications of lupus. LN occurs when SLE affects the kidneys, leading to a range of pathological changes in the renal tissues. The immune system's production of autoantibodies and deposition of immune complexes in the glomeruli can cause significant inflammation, resulting in varying degrees of glomerulonephritis. If left untreated, LN can progress to end-stage renal disease (ESRD), underscoring the need for early diagnosis and aggressive management. Treatment typically involves immunosuppressive therapies aimed at reducing inflammation and controlling the autoimmune response [25], [26], [27].

Rheumatoid arthritis (RA), a chronic inflammatory disorder primarily affecting the joints but can also involve other organ systems, including the kidneys. Renal involvement in RA may manifest as secondary amyloidosis, glomerulonephritis, or drug-induced nephropathy, the latter resulting from the long-term use of non-steroidal anti-inflammatory drugs (NSAIDs) and certain disease-modifying antirheumatic drugs (DMARDs) used in RA treatment. RA-associated interstitial nephritis and renal amyloidosis (AA amyloidosis) are among the conditions that can lead to CKD in RA patients. The management of RA-related kidney disease focuses on controlling the underlying RA activity and avoiding nephrotoxic medications [28].

The management of CKD secondary to autoimmune diseases focuses on controlling the autoimmune activity to prevent further kidney damage. This may involve the use of corticosteroids, immunosuppressants (e.g., cyclophosphamide, methotrexate, mycophenolate mofetil), and biologic agents tailored to the specific autoimmune condition and its severity. Antibody treatment has become a cornerstone in the management of autoimmune diseases, leveraging the precision of monoclonal antibodies to target specific molecules involved in the immune response. These therapies work by selectively inhibiting the activity of immune cells or blocking cytokines, which are substances that contribute to inflammation and tissue damage. For example, monoclonal antibodies such as infliximab and adalimumab target tumor necrosis factor-alpha (TNF-α), a key pro-inflammatory cytokine. Interestingly, testosterone downregulated TNF-α by reducing its production in a rabbit model [36].

Another example is rituximab, which targets CD20 on B cells, thereby reducing the production of autoantibodies. These targeted approaches offer the advantage of reducing systemic immunosuppression and minimizing side effects, providing a more tailored and effective treatment option for patients. Additionally, supportive treatment to manage CKD symptoms and complications, including hypertension, edema, and electrolyte imbalances, is crucial [23].

In conclusion, autoimmune diseases like SLE and RA can significantly impact renal function, contributing to the development and progression of CKD. Early recognition and comprehensive management of these conditions are essential to prevent CKD progression and preserve kidney function.

## Definition and stages of CKD

CKD is defined by the persistence of kidney structure or function abnormalities for more than three months, with implications on overall health. The diagnostic criteria for CKD include evidence of kidney damage (manifested by proteinuria, abnormalities in blood or urine tests, or imaging studies) or a decreased glomerular filtration rate (GFR), a key indicator of kidney function.

The progression of CKD is categorized into five stages based on the GFR, which reflects the extent of kidney function from normal to complete failure. This classification system aids in the diagnosis, management, and therapeutic planning for CKD, highlighting the disease's progression.

Stage 1 (GFR≥90 mL/min/1.73 m^2^) is characterized by a normal or high GFR alongside evidence of kidney damage. Signs of kidney damage may include proteinuria, hematuria, or anatomical changes detected on imaging studies. Management focuses on mitigating risk factors, addressing underlying conditions, and monitoring to prevent disease progression. Stage 2 (GFR 60–89 mL/min/1.73 m^2^) means a mild reduction in GFR with signs of kidney damage. Kidney function is slightly below normal, with structural or functional abnormalities present. The approach involves evaluating causative factors, managing comorbid conditions, and monitoring to slow disease progression. Stage 3 (GFR 30–59 mL/min/1.73 m^2^) is a moderate reduction in GFR, further divided into stage 3a (GFR 45–59 mL/min/1.73 m^2^) and stage 3b (GFR 30–44 mL/min/1.73 m^2^). This stage marks a critical point for intervention, focusing on controlling complications such as anemia, bone disease, hypertension, and cardiovascular risk. Symptoms related to CKD may begin to emerge. Stage 4 (GFR 15–29 mL/min/1.73 m^2^) represents a severe reduction in GFR. Interventions at this stage include advanced planning for kidney replacement therapy (dialysis or transplantation), addressing complications from reduced kidney function, and intensive management of health issues associated with CKD. Stage 5 (GFR<15 mL/min/1.73 m^2^) or on dialysis refer to kidney failure or end-stage renal disease (ESRD). Kidneys have lost nearly all their functionality, necessitating kidney replacement therapy (dialysis) or a kidney transplant to sustain life [24], [22], [23].

The evaluation and progression through CKD stages are determined by blood tests for GFR, urine tests for kidney damage markers, and imaging studies for structural abnormalities. The management of CKD aims at slowing the disease's progression, treating complications, and preparing for kidney replacement therapy when necessary. Early detection and intervention are pivotal in enhancing outcomes for individuals with CKD.

## General treatment options in CKD

The general approaches to treatment of chronic kidney disease (CKD) are multifaceted, aiming to slow disease progression, manage complications, and address the underlying causes. With advancements in medical research, newer treatment approaches have been developed. Here, I will highlight the current treatment strategies for CKD, including recent advancements up to the present [22], [23].

Controlling hypertension is critical in CKD management to slow progression. Angiotensin-converting enzyme inhibitors (ACEIs) and angiotensin receptor blockers (ARBs) are standard treatments due to their proven efficacy in reducing proteinuria and slowing CKD progression [37]. For CKD patients with diabetes mellitus, maintaining optimal glycemic control is essential to prevent progression. Sodium-glucose cotransporter 2 (SGLT2) inhibitors have emerged as a significant advancement, demonstrating benefits in reducing CKD progression and cardiovascular events in diabetic and non-diabetic CKD patients [38]. Proteinuria is a marker of kidney damage and a target for CKD treatment. Besides ACEIs and ARBs, newer agents like SGLT2 inhibitors and the nonsteroidal mineralocorticoid receptor antagonist finerenone have shown promise in reducing proteinuria and slowing CKD progression, offering additional benefits beyond traditional treatments [22], [23]. Dietary interventions, including reduced intake of sodium and protein, play a crucial role in managing CKD. The emphasis on plant-based diets has gained attention for their potential to slow CKD progression and improve outcomes. Regular physical activity and smoking cessation are also recommended [22], [23]. Anemia management in CKD has evolved with the development of hypoxia-inducible factor prolyl hydroxylase inhibitors (HIF-PH inhibitors). These agents stimulate endogenous erythropoietin production, offering an alternative to erythropoiesis-stimulating agents (ESAs) with the potential for fewer cardiovascular risks [39], [22], [23].

Treating CKD-associated mineral and bone disorder includes phosphate binders, vitamin D analogs, and calcimimetics. The choice of agents is tailored to the patient’s phosphate, parathyroid hormone, and calcium levels, aiming to prevent bone disease and vascular calcification. Advanced CKD and Kidney Replacement Therapy are for patients progressing to end-stage renal disease (ESRD) and involve dialysis (hemodialysis or peritoneal dialysis) and kidney transplantation, which are the mainstays of treatment. Preemptive kidney transplantation, when possible, is preferred due to better outcomes compared to dialysis [40], [41]. Emerging treatments for CKD are focusing on targeting specific pathways involved in kidney damage, including endothelin receptor antagonists, glucagon-like peptide-1 (GLP-1) agonists, and novel anti-inflammatory agents. Ongoing research and clinical trials continue to explore these and other potential treatments to offer hope for more effective CKD management in the future [22], [23].

The treatment of CKD requires a comprehensive approach that addresses the underlying causes, slows disease progression, and manages complications. Recent advancements, particularly in the areas of glycemic control, management of proteinuria, and anemia treatment, have significantly improved the therapeutic landscape, offering perspectives for patients with CKD.

## Why the link between testosterone deficiency and chronic kidney disease is important

The exploration of the link between testosterone deficiency and chronic kidney disease (CKD) is of paramount significance for several compelling reasons. Firstly, testosterone deficiency is prevalent among men with CKD, suggesting a bidirectional relationship where each condition potentially exacerbates the other [1]. One of the major endocrine disorders associated with CKD is male hypogonadism, which has a prevalence of 27 % to 66 % in this patient population [1], [42]. Low testosterone levels have a negative effect on the prognostic survival in men with CKD, albeit that does not allow the conclusion that TRT would have the potential to ameliorate this effect, as the causalities between CKD and male hypogonadism are multifactorial [1]. Understanding the mechanisms underlying the association described above could illuminate the pathophysiological pathways shared by metabolic, cardiovascular, and renal health, offering insights into holistic approaches to patient management [43].

Testosterone plays a crucial role in the regulation of muscle mass, bone density, erythropoiesis, and overall energy homeostasis [2]. In the context of CKD, where patients frequently suffer from muscle wasting, anemia, and alterations in mineral metabolism, the impact of testosterone deficiency can profoundly affect disease progression, quality of life, and mortality risk. Clarifying the extent to which testosterone supplementation could mitigate these complications presents a potential therapeutic avenue warranting rigorous investigation.

Moreover, testosterone has been shown to exert significant effects on renal function directly, including modulation of renal blood flow and filtration, anti-inflammatory actions, and potential interactions with pathways involved in fibrosis and progression of kidney damage [44], [10]. Investigating these effects may provide novel insights into the progression of CKD and uncover new targets for intervention. Real-world approaches have demonstrated positive outcomes on renal function in men with male hypogonadism [30], [45].

Older reviews indicate that the effects of sex steroids on different parts of the renal-vascular system may be complex and often contradictory [46], [11]. The potential mineralocorticoid properties of testosterone, including its ability to bind to the aldosterone receptor and influence blood pressure and fluid balance, reveal a complex interplay between hormonal regulation and renal function [5]. Thus, testosterone may be putatively associated with arterial hypertension through a hypothesized stimulation of the renin-angiotensin-aldosterone-system (RAAS), increased renal sodium reabsorption, and/or increased vascular resistance from amplified vascular smooth muscle cell proliferation. These mechanisms may ultimately result in arterial hypertension-mediated renal injury but remain speculative; they were shown in female rat models and are speculated to exist in human females [5], [47]. Contradictingly, in men, short-term testosterone application led to a decrease of serum aldosterone concentrations [48].

In addition, testosterone has been demonstrated to downregulate mRNA levels of aldosterone synthase, leading to decreased plasma aldosterone levels [15]. Furthermore, androgens have been shown to act as potential antagonists of the mineralocorticoid receptor [49]. In fact, a rat model revealed that endogenous androgens exerted anti-hypertensive effects that appear to involve non-genomic and possibly genomic mechanisms, resulting in reductions in RAAS expression and enhanced systemic vasodilation [20]. A recent paper showed that men of a large cohort with total testosterone serum levels < 213 ng or < 7.4 nmol and these with low estradiol serum levels have an increased all-cause mortality including cardiovascular disease [19].

Currently, there is no known human model, such as mutants with androgen receptor modulation exhibiting less responsiveness or resistance and consequently higher total testosterone levels, that demonstrates increased stimulation or inhibition of the RAAS and/or mineralocorticoid receptor leading to elevated or decreased blood pressure.

However, testosterone might also directly induce renal tubule cell injury by activating the Fas-FasL mediated apoptosis pathway, which is inhibited when estradiol is present [7]. Testosterone is also speculated to exert additional oxidative stress on kidneys and lead to excessive extracellular matrix deposition [6].

In sum, studying the link between testosterone deficiency and CKD not only enhances understanding of the intricate interactions between endocrine and renal health but also offers potential pathways for developing targeted therapeutic strategies that address the multifaceted needs of patients suffering from these interconnected conditions.

## Conjunct epidemiology of testosterone deficiency and chronic kidney disease

Epidemiological evidence increasingly indicates a higher prevalence of testosterone deficiency among patients with chronic kidney disease (CKD), compared to the general population. This relationship has been documented across various stages of CKD, including those not on dialysis, those undergoing dialysis, and kidney transplant recipients. The mechanisms behind this association are multifactorial, involving the complex interplay between the decline in kidney function, changes in hormone metabolism, and the systemic effects of CKD. Studies have consistently shown that as kidney function declines, the prevalence of testosterone deficiency increases. Low testosterone levels are common in men with CKD and associated with a higher risk of death, independent of other risk factors [1]. This *meta*-analysis, involving 28,663 men with CKD and controls, found that those with the lowest testosterone levels had significantly higher mortality rates, underscoring the potential impact of testosterone deficiency on patient outcomes [1].

Also vice-versa, low serum testosterone levels were associated with a higher incidence of CKD among male adults in a prospective population-based study: During a 15-year follow-up study, a total of 1277 eligible male adults aged 20–80 years consisting of 605 males with low testosterone levels (<350 ng/dL) and 672 controls with normal levels were recruited. A higher hazard ratio of CKD progression in male adults with hypogonadism compared to those with normal levels in their later life was observed [50].

A study in patients in a pre-dialysis state highlighted that testosterone deficiency was not only prevalent but also associated with worse outcomes, including cardiovascular events and mortality. This study suggested that the inflammatory state and malnutrition commonly seen in CKD could contribute to the observed male hypogonadism [51]. Testosterone deficiency was also a cause of anemia and reduced responsiveness to erythropoietin [52]. In patients with ESRD being on hemodialysis, low testosterone levels did not act as a major predictor of mortality but were associated with deteriorated surrogate markers, i.e., lower scores of the HUI3 (Health Utilities Index Mark 3) and KDQOL-12 PCS (Kidney Disease Quality of Life-12 Physical Component Summary). Also, the prevalence of concomitant cardiovascular disease was higher in men with low serum concentrations of testosterone [53].

The pathophysiological mechanisms linking CKD with testosterone deficiency include uremic toxins: Accumulation of uremic toxins in CKD can impair Leydig cell function, leading to reduced testosterone synthesis. CKD is also associated with chronic inflammation, which can suppress the hypothalamic-pituitary–gonadal (HPG) axis, further contributing to low testosterone levels. Furthermore, malnutrition, common in advanced CKD, can negatively affect testosterone production. The clinical implications of testosterone deficiency in CKD are significant. Testosterone plays a crucial role in muscle mass, bone density, erythropoiesis, and overall quality of life. Deficiency in testosterone can contribute to the muscle wasting, anemia, and bone disease often seen in CKD patients, thereby exacerbating morbidity and potentially influencing mortality [2].

The epidemiological evidence clearly indicates a strong association between testosterone deficiency and CKD, with significant implications for patient outcomes. Understanding this relationship is crucial for the holistic management of CKD patients, emphasizing the need for routine screening for testosterone deficiency as part of comprehensive care in this population.

## Mechanisms of testosterone deficiency due to chronic kidney disease

Male hypogonadism in the context of chronic kidney disease (CKD) represents a multifaceted clinical issue, where the interplay between reduced kidney function and hormonal imbalances culminates in a state often referred to as “secondary” or “functional” male hypogonadism. The pathophysiology underlying this condition is complex, involving various factors related to the systemic effects of CKD on the body's endocrine functions. It is a paradigm that illustrates how a chronic systemic disease can disrupt hormonal regulation and synthesis, leading to both primary and secondary male hypogonadism.

Chronic kidney disease (CKD) has a complex relationship with male hypogonadism, with several potential mechanisms at play that link the two conditions. As CKD progresses, the kidneys' ability to filter waste efficiently diminishes, leading to an accumulation of uremic toxins. These toxins adversely affect the Leydig cells in the testes, which are crucial for testosterone production, resulting in a type of primary male hypogonadism. The interference does not stop there; uremic toxins also disrupt the hypothalamic-pituitary–gonadal (HPG) axis, further hampering the synthesis of testosterone [42], [1]. A mixed picture of primary and secondary male hypogonadism, resembling the concept of functional male hypogonadism, at least partly, might result from the influence of CKD on the hypothalamic-pituitary–gonadal axis.

Additionally, CKD often triggers chronic inflammation, a condition marked by elevated levels of inflammatory cytokines such as TNF-α and IL-6. These cytokines are known to suppress the HPG axis, reducing the secretion of gonadotropin-releasing hormone (GnRH) from the hypothalamus and subsequently decreasing the production of luteinizing hormone (LH) and follicle-stimulating hormone (FSH) from the pituitary gland. This cascade of effects significantly impacts testosterone levels [54], [55], [56], [42], [1]. Vice versa, testosterone can mitigate a pro-inflammatory state in hypogonadal men [13].

Nutritional deficiencies present another link between CKD and male hypogonadism. Patients with CKD often experience malnutrition and vitamin D deficiency, which negatively affect testosterone synthesis. This may be exacerbated by increased visceral fat and hyperproduction of leptin, which together affect both Leydig cell function and the hypothalamic-pituitary axis' ability to secrete gonadotropins. This nutritional imbalance is particularly detrimental for men undergoing hemodialysis, contributing to muscular atrophy and physical inactivity [57], [58].

Anemia is another frequent complication in CKD patients that influences testosterone levels. The use of erythropoietin (EPO) therapy to treat anemia in CKD has been suggested to suppress the HPG axis as well [59]. This introduces a complex interplay where treatment for one symptom of CKD inadvertently impacts another crucial hormonal pathway.

Finally, CKD modifies the metabolism of sex hormone-binding globulin (SHBG), a protein that binds to testosterone, thus affecting its bioavailability. Changes, i.e., increases, in SHBG levels can lead to decreased circulating free testosterone—the biologically active form of the hormone. Such altered metabolism, showcasing how CKD leads to a reduction in available testosterone, was demonstrated [60].

These interconnected pathways illustrate the multifaceted relationship between CKD and male hypogonadism, underscoring the importance of a comprehensive approach to managing these patients that considers both renal function and hormonal balance.

## Male hypogonadism in End-Stage renal disease

The intricate interplay between hormonal function and kidney health is elucidated in three recent studies, each providing significant insights into endocrine disturbances in patients undergoing hemodialysis and kidney transplantation.

The relationship between serum testosterone concentrations and clinical outcomes in hemodialysis patients was explored within the EVOLVE trial. Findings underscore the critical role of testosterone as a biomarker for morbidity and mortality in this population. The study highlights that lower testosterone levels are prevalent among hemodialysis patients and are associated with adverse outcomes, including increased cardiovascular events and overall mortality. The mechanistic underpinnings of these observations suggest that testosterone deficiency may exacerbate the already heightened inflammatory state and oxidative stress characteristic of end-stage renal disease (ESRD), further compromising patient health [37].

A comprehensive review of thyroid, gonadal, and adrenal dysfunction in kidney transplant recipients delineates the multifaceted endocrine alterations post-transplant, emphasizing that thyroid dysfunction, male hypogonadism, and adrenal insufficiency are prevalent and contribute to significant clinical challenges. An intricate balance is required in managing these hormonal disorders to optimize transplant outcomes and enhance patient quality of life. Emphasis is made on the necessity for vigilant monitoring and tailored therapeutic strategies to address these endocrine abnormalities, thereby reducing the risk of metabolic complications and improving long-term graft survival [38].

A focus on the levels of sex hormone-binding globulin (SHBG) and the relationship with insulin resistance in men undergoing chronic hemodialysis was the subject of another research. Elevated SHBG levels were correlated with greater insulin resistance, suggesting a potential compensatory mechanism or a marker of underlying metabolic derangements. This study highlights the importance of considering hormonal profiles in managing metabolic syndrome in hemodialysis patients, advocating for a more integrated approach to treatment that encompasses endocrine evaluation and intervention [39].

Together, these studies paint a comprehensive picture of the hormonal challenges faced by patients with chronic kidney disease and those undergoing renal replacement therapies. They underscore the importance of holistic and individualized patient care that addresses the multifaceted endocrine disturbances to improve outcomes and quality of life in this vulnerable population, also beyond male hypogonadism.

## Historic use of testosterone in CKD to treat anemia

Before the advent of erythropoietin as a therapeutic agent, testosterone was extensively utilized in the management of anemia in dialysis patients with end-stage renal disease (ESRD). In the early 1970 s, clinicians recognized the hematopoietic properties of testosterone, which significantly ameliorated the anemic conditions in these patients. Anemia in patients with CKD was one of the former indications to use also other androgenic anabolic steroids as official medication [61], [62], [63]. The efficacy of TRT was elucidated in combining iron supplementation with androgen therapy to enhance hemoglobin levels in periodic hemodialysis patients, underscoring testosterone's ability to stimulate erythropoiesis and compensate for the impaired endogenous erythropoietin production [64]. Similarly, substantial improvements in hematocrit and red blood cell counts in maintenance dialysis patients treated with testosterone were reported, thus demonstrating its pivotal role in managing renal anemia before erythropoietin became available [65].

Subsequent studies further elucidated the mechanisms underlying testosterone's efficacy in treating renal anemia. Morphometric bone marrow and erythrocyte volume assessments were employed to quantify the hematopoietic response to testosterone therapy, confirming its significant impact on erythropoiesis in ESRD patients [66]. This foundational research was corroborated by providing a comprehensive analysis of androgen application in anemia associated with chronic renal failure. Such findings reinforced earlier observations, solidifying testosterone's role as an essential precursor to erythropoietin therapy in managing ESRD-associated anemia [67]. Collectively, these studies not only highlighted the critical need for effective anemia management strategies in dialysis patients but also paved the way for the development of erythropoietin as a targeted effective treatment modality.

## Potential benefits and risks of testosterone therapy in chronic kidney disease

Testosterone therapy (TRT) in chronic kidney disease (CKD) patients presents a nuanced landscape of potential benefits and risks, demanding careful consideration. Here's an overview of how TRT might affect CKD patients, underlining its therapeutic prospects alongside cautionary notes.

## Potential benefits of testosterone therapy in CKD patients

TRT has been shown to enhance muscle mass and strength, which can be particularly beneficial for CKD patients who often experience muscle wasting and reduced physical function [2]. Improved muscle strength can enhance the quality of life and potentially reduce the risk of falls and fractures. In autoimmune-induced CKD, TRT might exhibit benefits via reducing the load of inflammatory substances [13]. It might as well improve the diabetic and inflammatory state in diabetes-induced CKD [68].

Testosterone stimulates erythropoiesis, which can help address the anemia commonly seen in CKD patients [16], [17]. Contributingly, testosterone deficiency is a cause of anemia and reduced responsiveness to erythropoiesis-stimulating agents in men with chronic kidney disease [52]. Additionally, by improving bone density, TRT may mitigate the risk of osteoporosis [10], [69], a condition prevalent in the CKD population due to altered mineral metabolism and vitamin D deficiency. Notwithstanding, patient studies within this cohort are lacking. In a rat model of CKD, a benefit on bone density induced by testosterone administration was not seen [70].

Some evidence suggests that TRT might offer cardiovascular benefits by improving body composition, insulin sensitivity, endothelial function, inflammatory status, and lipid profiles in hypogonadal men with type 2 diabetes mellitus [68], [71], [45], [12], potentially resulting in decreased mortality versus untreated hypogonadal men with diabetes [72]. The largest randomized controlled trial on testosterone therapy involving more than 5000 older hypogonadal men did not exhibit an increased rate of cardiovascular events in these subjects with already established cardiovascular disease [14]. A trial conducted specifically in men with CKD and male hypogonadism as well as diabetes mellitus does not exist to date. Nevertheless, 15–16 % of the population of the above-named trial had stage 3 CKD.

TRT can stimulate prostate tissue growth, which has raised concerns about the potential exacerbation of benign prostatic hyperplasia (BPH) or the risk of prostate cancer in the past. The above-mentioned largest randomized trial on TRT to date did not show an increased risk for the development of prostate cancer in older men [2]. In this trial, a slightly higher rate of “kidney injuries” was seen, without any further definition of these events [14].

Testosterone therapy (TRT) in hypogonadal men with CKD has the potential to significantly improve sexual wellbeing, general mood, and physical abilities, providing benefits that extend beyond its possible effects on CKD. In this regard, men with CKD and male hypogonadism have to be seen as hypogonadal patients as such. TRT has been shown to enhance libido, erectile function, and overall sexual satisfaction, addressing common issues of reduced sexual desire and performance that are prevalent in men with low testosterone levels. In terms of mood, testosterone plays a critical role in regulating psychological health, with deficiencies often linked to depression, irritability, and cognitive decline. By restoring testosterone to normal levels, TRT can improve mood, enhance cognitive function, and reduce the risk of depression, thereby contributing to better mental health and overall wellbeing. Furthermore, testosterone is crucial for muscle protein synthesis and bone density maintenance. TRT can lead to increased muscle mass and strength, improved physical performance, and greater energy levels, enabling men to engage more actively in daily activities and exercise, thus enhancing their quality of life. These comprehensive benefits underscore the importance of TRT in addressing the multifaceted health challenges faced by hypogonadal men [2], [9], [21]. This also applies to patients with CKD, albeit with a special emphasis on whether TRT might affect their renal disorder in a positive or adverse manner.

## Potential risks of testosterone therapy in CKD

**Fig. 3:**
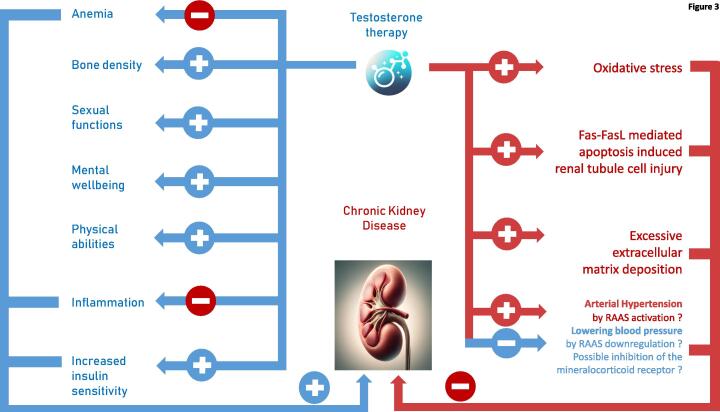
Putative effects of Testosterone Therapy in Hypogonadal men with CKD including beneficial effects on symptoms related to CKD as being caused by it and potential impact of favorable or adverse nature on the progression of CKD itself. The nature of this diagram remains speculative and is, regarding alleviation of clinical symptoms, derived from knowledge about general testosterone effects in male hypogonadism. The assumed adverse affections of CKD by testosterone are also more or less hypothetical as they are based on associations or cell models. RAAS: Renin-Angiotensin-Aldosterone-System.

The effects of TRT on CKD progression are not fully understood. There is a concern that fluid retention associated with TRT might exacerbate arterial hypertension via direct binding of testosterone to the aldosterone receptor or activation of the RAAS, resulting in heart failure in susceptible individuals, potentially impacting the progression of renal diseases. This might also be facilitated by enhancing renal tubule cell injury via augmented apoptosis [7], [5], [6]. Such assumptions remain speculative, to date. See Fig. 3 for Summary.

## Recommendations for testosteron therapy in men with chronic kidney disease

Given the complex interplay of benefits and risks, the following topics are suggested to be considered for TRT in CKD patients, in principle not diverting much from general recommendations on TRT:

The patient should be clearly hypogonadal with symptoms according to guidelines [2].
Monitoring: Regular monitoring of testosterone levels, hematocrit, prostate-specific antigen (PSA), and cardiovascular health is essential. Adjustments to TRT should be made based on clinical response and side effects [2].
As muscle mass will increase during testosterone therapy, so might serum creatinine. This has to be taken into consideration. An increase of serum creatinine might, in this case, not clearly reflect an advancing renal damage. It is recommended to determine muscle-independent markers such as cystatin C to determine kidney function [73]. Cystatin C-based calculations have been demonstrated to provide a better prediction of the progression of CKD and its outcome, also mortality, especially in elderly cohorts [74], [75], [76].
Consider TRT on a case-by-case basis, evaluating the severity of male hypogonadism symptoms, baseline testosterone levels, and individual risk factors. Genetic variations in CYP3A4 and SRD5A2, crucial for testosterone metabolism, can influence TRT efficacy. Adjusting TRT dosages based on these genetic profiles could enhance treatment outcomes [77]. Variants in the androgen receptor gene impact receptor sensitivity, suggesting a need for personalized TRT plans to optimize effectiveness [55], [78], [16], [79].
Contraindications: Avoid TRT in CKD patients with a history of prostate or breast cancer, severe sleep apnea, uncontrolled heart failure, or a recent history of myocardial infarction or stroke, as well as wish for paternity [2], [21].
Multidisciplinary Management: Collaboration among nephrologists, andrologists, urologists, endocrinologists, and also primary health care providers can optimize patient outcomes, balancing the benefits of TRT with its potential risks.

In conclusion, while TRT could offer significant benefits for CKD patients with male hypogonadism, including improvements in muscle mass, anemia management, decreasing inflammation, and potentially improving cardiovascular health in a diabetic subpopulation, it may not be without risk in these men, especially those exhibiting uncontrolled hypertension. Careful patient selection, monitoring, and a multidisciplinary approach are crucial to harness the benefits of TRT while minimizing its risks.

## Author statement

I am pleased to submit my invited revised review article for consideration in the Journal of Clinical and Translational Endocrinology. This review comprehensively examines the intricate relationship between testosterone levels and chronic kidney disease (CKD), focusing on the bidirectional interactions, clinical implications, and potential therapeutic approaches.

I declare that I have written the text myself and without help from other persons.

## CRediT authorship contribution statement

**Michael Zitzmann:** Writing – review & editing, Writing – original draft, Visualization, Validation, Supervision, Methodology, Investigation, Formal analysis, Data curation, Conceptualization.

## Declaration of competing interest

The authors declare that they have no known competing financial interests or personal relationships that could have appeared to influence the work reported in this paper.
